# A Deeper Understanding of Physics, Physiology, Experimental Methodology, and Statistics is Essential for Valid Comparison of USCOM 1A and cMRI

**DOI:** 10.1007/s00246-021-02604-2

**Published:** 2021-04-20

**Authors:** Rob Phillips, Joe Brierley

**Affiliations:** 1 Cardiovascular Physiology and Monitoring, Critical Care Research Group, School of Medicine, The University of Queensland, Brisbane, Australia; 2 Cardiovascular Physiology and Critical Care, Jining University Medical School, Peoples No1 Hospital, Jining, Shandong China; 3 Paediatric and Neonatal Intensive Care Unit, Great Ormond Street Children’s Hospital, London, UK

Weissbach et al. [1] make a number of assumptions related to cardiovascular physiology, physics, experimental methodology, and statistics which impair the strength of their conclusions in comparing the USCOM 1A digital Doppler ultrasound and cMRI for measurement of hemodynamics in adolescents with heart disease.

The continuity equation and principles of conservation of mass require flow and cross-sectional area (CSA) measures be made at the exact same location for accurate calculation of flow volumes. The aortic valve annulus (AVA), sinuses of Valsalva (SOV), and ascending thoracic aorta (ATA) are sequential sections of the aorta with different CSA diameters, and distinct flow and pressure profiles, which respond distinctly to changes in physiology, disease, and therapy. The USCOM 1A appropriately combines the flow and CSA at the AVA. However, the cMRI method used for this study employs a hybrid method combining flow from the ATA, beyond the sino-tubular junction, with the “LVOT” measured as “diameter of the aortic sinuses (SOV) … obtained in systole, with the largest diameter used in the calculation,” a method not compliant with the continuity equation. As the normal SOV is 16% greater in diameter than the ATA [2], this generates a potential 34% error in cMRI flow volume. Additionally the SOV differs by 54% from the AVA [2], the site of USCOM 1A measures, thus invalidating both the cMRI measurement of flow, and the comparison with the USCOM 1A.

Importantly, both the SOV and ATA used in the cMRI calculations are elastic and act as pressure reservoirs distending and contracting during the cardiac cycle, thus imposing intracyclic variations in CSA which may exceed 20% [3]. Respiration further varies normal flow by ± 12% [4], as do changes in blood pressure (BP) [5], with both impacting regional pressure and ATA diameter. These variables, although both significant, were not discussed nor corrected for in the cMRI flow volume calculations. The site of the USCOM 1A CSA, the AVA, is bedded in the fibrous base of the heart and is effectively inelastic regardless of output, BP, respiration, and across each cardiac cycle [3].

Significantly the flow measured by cMRI is beyond the take-off of the coronary arteries, which represent approximately 5% of output in the resting subject, and up to 25% during exercise, disease, and therapy [6], thus adding an additional source of non-linear error to the measurement. Further the flow in the ATA is helical and highly variable according to individual thoracic aortic morphology, including the position and angle of the vascular take-offs [7], which may further compromise the ATA as a site for flow measurement.

The cMRI method acquired magnetic data from 2 separate signal acquisition series, plus an averaged magnetic flow series of approximately 30 heart rate phases in free breathing subjects. This free breathing creates a 1–2-cm excursion of the thoracic aortic as it moves through the fixed magnetic beam, effectively translating the ATA flow sample volume with each respiration. Therefore, the flow and CSA measures by the cMRI were from different levels of the aorta, at different times as the target moved through the sample volume, over 30 cardiac cycles, thus ensuring the loss of much of the clinical sensitivity of an accurate beat-to-beat method. The USCOM 1A uses digital Doppler ultrasound to measure the flow of red blood cells at the level of the AVA prior to the coronary take-offs where flow is complete and most laminar and is instantaneous and beat to beat. Comparing the same parameter at the same location at the same time is vital for experimental accuracy.

The cMRI magnetic data were estimated to have a 10–20-min acquisition time and a further 60 min for remote generation of a single CO measurement using proprietary algorithms on an offline engineering workstation. Such a single daily measure of average hemodynamics is of low clinical utility in an environment where beat-to-beat analysis of SV provides the critical insights into the hemodynamics of cardiovascular function, fluid status, the response to therapy, and death. The USCOM 1A generates real-time SV’s at a sample rate of 100fps displaying a beat to beat readout of 26 hemodynamic parameters, with an entire examination completed in less than 5 min; less than the time to boot the cMRI.

The Bland–Altman (BA) method of 1986 used for this study was devised to compare equal measures of the same variable measured at the same time by different methods, preconditions absent from the current study. The cMRI values represent 30 heart cycles of averaged flow data collected over an unreported time integrated with morphologic data collected at a different time, compared to a much smaller sample from the USCOM 1A acquired over a few seconds and collected after a further delay at a different location. BA published revised methods to specifically deal with such imperfect comparisons in 1999 [8], and then in 2007 [9], but neither were adopted for this study.

Further the study conclusion is predicated on the assumption that cMRI is a reference standard for the BA comparison. However, BA only demonstrates agreement, not accuracy, and the choice of cMRI as the reference method when compared with the established evidence and clinical adoption of USCOM 1A is unjustifiable [10].

The application of cMRI as a hemodynamic tool is limited by its uncertain accuracy, high capital and personnel costs, inaccessibility, poor temporal resolution, and its limited application in subjects on ventilation, with dysrhythmias, and dynamic pathological, pathophysiologic, or therapeutic responses, precisely the patients in whom advanced hemodynamics is critical.

Comparison of methods requires a deeper understanding of physics, physiology, experimental methodology, and statistics to form a valid scientific conclusion. While cMRI may be an interesting nascent tool, the current study does not justify the enthusiastic conclusions of the authors.
